# Association between alpha-thalassaemia trait, *Plasmodium falciparum* asexual parasites and gametocyte carriage in a malaria endemic area in Southern Ghana

**DOI:** 10.1186/s13104-019-4181-8

**Published:** 2019-03-13

**Authors:** Helena Lamptey, Michael Fokuo Ofori, Bright Adu, Kwadwo Asamoah Kusi, Emmanuel Kakra Dickson, Isabella Quakyi, Michael Alifrangis

**Affiliations:** 1 Immunology Department, Noguchi Memorial Institute for Medical Research, College of Health Sciences, University of Ghana, Legon, Ghana; 2 Department of Biological, Environmental and Occupational Health Sciences, School of Public Health, College of Health Sciences, University of Ghana, Legon, Ghana; 3 Centre for Medical Parasitology, Department of Immunology and Microbiology, University of Copenhagen, Copenhagen, Denmark; 4 Department of Infectious Disease, National University Hospital (Rigshospitalet), Copenhagen, Denmark

**Keywords:** *Plasmodium falciparum*, Submicroscopic parasites, Alpha-thalassaemia, Gametocyte carriage

## Abstract

**Objective:**

The alpha-thalassaemia trait has been associated with protection against severe malaria but its role in *Plasmodium falciparum* asexual parasite and gametocyte carriage remains unclear. This study examined association between prevalence of α-thalassaemia and *P. falciparum* asexual stage parasitaemia and gametocytaemia in children, pregnant women and adults, which was part of a bigger study that investigated some key factors that influence gametocyte carriage.

**Results:**

Overall prevalence of heterozygous α-thalassaemia trait among all the groups was 39.0%, while 8.2% were homozygous alpha thalassaemia. Asexual parasite prevalence was significantly higher in children (P = 0.008) compared to adults and pregnant women. Of the asexual *P. falciparum* positive individuals, gametocyte prevalence was 38.5% (15/39) in children, 29.7% (11/37) in pregnant women and 17.4% (4/23) in adults. Heterozygous α-thalassaemic children were less likely to harbour asexual parasites, compared with normal and those deficient (OR = 0.52; 95% CI 0.28–0.97; P = 0.037) under the dominant model. These heterozygous children were also associated with reduced risk of parasitaemia compared to heterozygous adults and pregnant women. Children with heterozygous α-thalassaemia trait had reduced risk of asexual parasite carriage. There was however, no association between α-thalassaemia trait and risk of gametocyte carriage.

## Introduction

Certain genetic blood disorders such as the thalassaemia’s (α, β) and sickle cell anaemia have been shown to influence malaria pathogenesis [1–3]. Heterozygous α–thalassaemia have been shown to confer protection against severe malaria [4], however, there is limited data on its effect on asymptomatic parasite carriage [5, 6]. Some studies have shown that certain genetic traits like sickle cell that protect against severe malaria could also enhance gametocyte carriage [1, 2]. Thus, these variants may promote sexual differentiation of the asexual parasites [1], as shown in previous in vitro studies in which reticulocyte-rich red cells of sickle cell variants enhanced higher gametocytes formation compared to the normal red cells [7, 8]. However, an association between α–thalassaemia and submicroscopic asexual parasites and gametocytes have, to the best of our knowledge, not been studied. Submicroscopic gametocytes have been shown to be important in malaria transmission [9, 10], therefore, knowledge on the effect of α-thalassaemia trait on submicroscopic asexual parasite and gametocyte carriage may be important for targeted control. However, studies examining such associations are limited and it is not clear whether these associations differ between different demographic groups, since children and pregnant women have been shown to be important gametocyte reservoirs and may impact on transmission [11–13]. This study determined asexual parasite and gametocyte carriage and explored possible association with α-thalassaemia among Ghanaian children, adults and pregnant women.

## Main text

### Methods

#### Study population and design

The study was conducted in Asutsuare and its surrounding villages in the Shai Osudoku District of Ghana. Baseline samples from a longitudinal study conducted from November 2013–September 2014 were used. Ethical approval was given by the Institutional Review Board of Noguchi Memorial Institute for Medical Research, University of Ghana, Legon. The study included three different demographic groups; children (aged 2–15 years), male and non-pregnant female adults (aged 16–65 years), and pregnant women (aged 18–45 years). Informed consent was obtained from all participants and/or their parents/legal guardians prior to their recruitment and inclusion in the study. A detailed description of the study area and population has been presented elsewhere [13].

#### Sampling techniques and laboratory procedures

All study participants were screened for their sickle cell status using the sodium metabisulphite method, and this grouped all SS and SC under AS phenotypes. Haemoglobin levels were measured using Hemocue-Hb 201 (Angelholm, Sweden). The ABO blood type was determined using a commercial anti-sera blood grouping kit (Biotec Laboratories Limited, UK).

Blood smears were stained with 10% Giemsa for detection of both asexual and sexual stage parasites by microscopy under oil immersion (100× magnification). Submicroscopic *P. falciparum* parasites were determined by a previously described nested PCR targeting sequences of the small subunit ribosomal (ssrRNA) 18 s genes of four *Plasmodium* species (*P. falciparum, P. malariae, P. ovale and P. vivax*) [14]. RNA was extracted from RNA-later preserved samples that were asexual parasite positive by microscopy and / or PCR using the RNeasy^®^ plus mini kit (Qiagen, Germany). Gametocyte detection was done by real-time (RT) quantitative PCR [15] targeting *Pf*s25 transcripts and RNA template for the reaction included 3D7 gametocyte-positive standard RNA generated from parasite culture as previously described [13].

Polymorphisms in the human α globin gene resulting in α-thalassaemia, specifically the—α 3.7 deletion, was determined by PCR [16]. After genotyping, participants were categorized as normal individuals, heterozygous α-thalassaemia or homozygous α-thalassaemia based on band size. For single marker association of α-thalassaemia alleles with *P. falciparum* infection, it was analyzed under three models of inheritance (dominant, additive and recessive) [17] using logistic regression and adjusting for age as a confounder.

### Results

#### Submicroscopic asexual parasite and gametocyte prevalence

The study population comprised of 463 subjects consisting of children (184), adults (154) and pregnant women (125), of which a total of 167 (36.1%) of the population were asexual parasite positive. Generally, majority of the parasites detected were submicroscopic. The distribution of asexual *P. falciparum* infections was significantly (P = 0.008, χ^2^-test) different among the study groups and majority of these infections were submicroscopic (97%). The prevalence was highest among the children (49.1%), compared to adults (28.7%) and pregnant women (22.2%) (Table 1). Only available samples that were positive for asexual parasites by PCR (n = 99) were further assessed for gametocytes. From these positives, the gametocyte prevalence was 38.5% (15/39) among the children, 17.4% (4/23) in the adults and 29.7% (11/37) in the pregnant women. The sickle cell trait distribution among the entire study population for all groups was 4.0%. There were no significant differences in haemoglobin levels, sickle cell trait and blood groups among individuals with parasites and those without parasites (P > 0.05), Table 1.

**Table 1:** Demographic and clinical characteristics of study population

Variables	Parasite	Parasite	Total Sample (n = 463)	P value
	Negative (n = 296)	Positive (n = 167)		
Groups (%)				
Children	102 (34.5)	82 (49.1)	184 (39.7)	0.008
Adults	106 (35.8)	48 (28.7)	154 (33.3)	
Pregnant women	88 (29.7)	37 (22.2)	125 (27.0)	
Gender (%)				
Female	215 (72.6)	122 (73.1)	337 (72.8)	1.0
Male	81 (27.4)	45 (26.9)	126 (27.2)	
Sickle cell trait (%)				
Negative	272 (95.8)	156 (96.3)	428 (96.0)	0.984
Positive	12 (4.2)	6 (3.7)	18 (4.0)	
Missing	12	5	17	
Blood group (%)				
A	57 (19.8)	30 (18.4)	87 (19.3)	0.517
AB	14 (4.9)	12 (7.4)	26 (5.8)	
B	93 (32.3)	45 (27.6)	138 (30.6)	
O	124 (43.1)	76 (46.6)	200 (44.3)	
Hemoglobin levels mean, (SD)	11.1 (1.7)	11.0 (1.6)	11.1 (1.7)	0.329

P values were calculated by Pearson’s Chi squared test. P value < 0.05 is significant

#### The distribution of alpha thalassaemia and association with parasite carriage

The overall prevalence of heterozygous α–thalassaemia was 39.0% (176/451) and 8.2% (37/451) carried the deficient homozygous trait. The distribution of α–thalassaemia alleles in the study population did not deviate from Hardy–Weinberg equilibrium (P = 0.56).

**Fig. 1:**
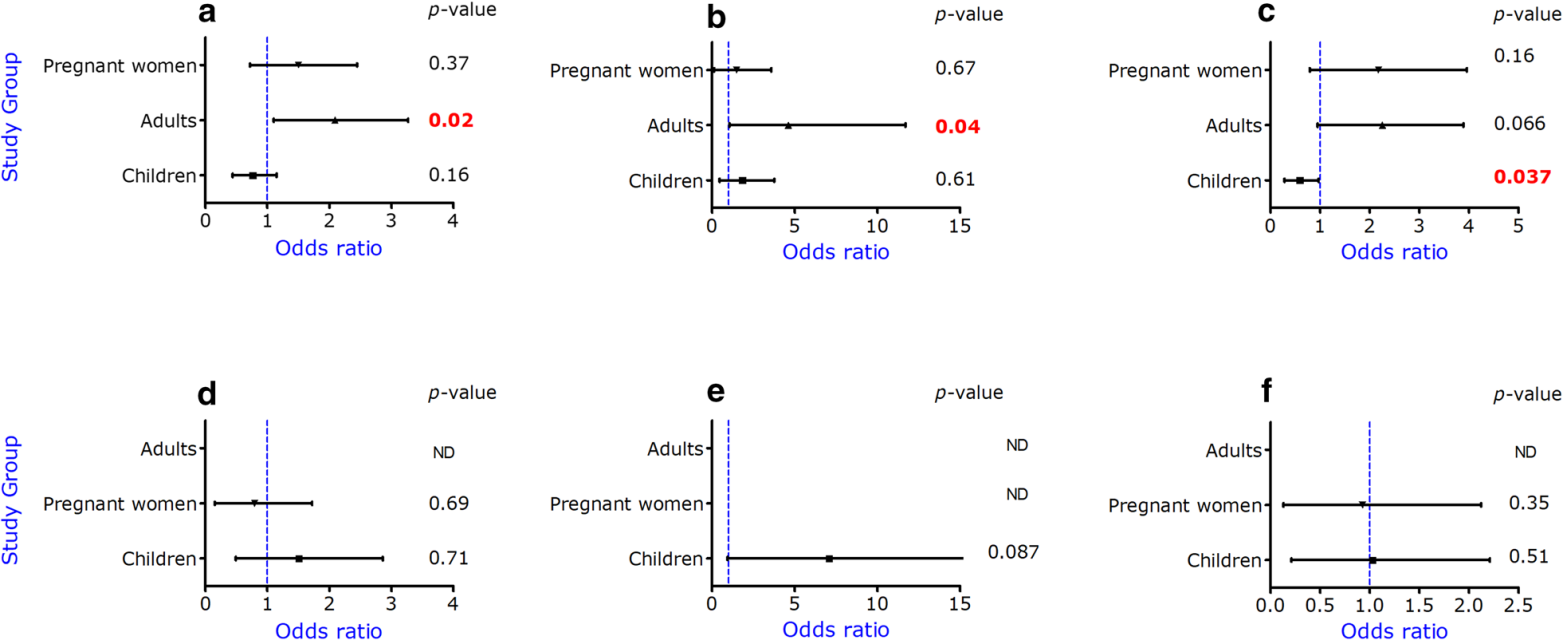
Alpha-thalassaemia association with asexual parasites (top row, **a**–**c**) and gametocytaemia (bottom row, **d**–**f**) among the groups. Three different models were used in a logistic regression analysis correcting for age. P value < 0.05 is significant. Plots represent additive model (**a** and **d**), recessive model (**b** and **e**) and dominant model (**c** and **f**) respectively

Overall, the heterozygous α-thalassaemia trait was not associated with asexual parasitaemia (P > 0.05, Table 2). However, when the analysis was done with individual groups, the heterozygous trait was associated with reduced risk of asexual parasitaemia in children (OR = 0.52; 95% CI 0.28–0.97; P = 0.037) but not in adults (OR = 1.92; 95% CI 0.95–3.89; P = 0.066) or pregnant women (OR = 1.7; 95% CI 0.79–3.9; P = 0.16) under the dominant model (Fig. 1a). With the additive and recessive models, heterozygous adults were more likely to carry asexual parasites, compared to the other groups (additive model: OR = 1.90; 95% CI 1.10–3.27; P = 0.02 and recessive model: OR = 1.10; 95% CI 1.05–11.69; P = 0.04) (Fig. 1, Top row). There was no significant association between α-thalassaemia trait and gametocytaemia in pregnant women and children as well as adults with all models (Fig. 1, bottom row).

**Table 2:** Single marker association of α-thalassaemia alleles with *P. falciparum* infection

Population	Additive model		Recessive model		Dominant model	
	OR (95% CI)	*P*-value	OR (95% CI)	*P*-value	OR (95% CI)	*P*-value
Asexual parasites	1.10 (0.81–1.49)	0.53	1.41 (0.71–2.81)	0.33	1.05 (0.71–1.56)	0.797
Gametocytes	0.88 (0.4–1.6)	0.67	1.42 (0.4–4.98)	0.60	0.71 (0.32–1.58)	0.40

Odds ratio (OR) and 95% confidence intervals (CI) were determined using multivariate logistic regression adjusting for age

P < 0.05 is significant

## Discussion

This study investigated associations between the α-thalassaemia trait and *P. falciparum* asexual parasitaemia and gametocytaemia in subjects of different demographic backgrounds in malaria endemic communities in southern Ghana. The overall distribution of heterozygous α–thalassaemia observed in this study area was relatively higher (20% to 30%) than observed in other parts of the country [4, 18, 19]. This was probably due to the fact that the community is populated with diverse ethnicity, hence there might have been a lot of inter marriages among the various tribes that might have influenced heterozygosity. Similarly, other studies conducted in other parts of Ghana, and elsewhere have reported a higher prevalence of the heterozygous alpha thalassaemia trait [20–22].

The association of asexual parasite carriage with α-thalassaemia trait varied among the groups. This difference in association may be due to possible confounding factors since protection from malaria has been shown to depend on several factors including the host’s immune status as well as host genetics [23]. In this study, children with heterozygous α-thalassaemia trait had two times reduced risk of harbouring asexual parasites under the dominant model, compared to the normal α-thalassaemic carriers after adjusting for age. This finding suggests that the reduced risk of asexual parasite carriage may decrease the likelihood of these children experiencing severe malaria disease. This is in agreement with studies that found heterozygous α-thalassaemia being associated with protection from severe disease [4, 6, 18]. Previous studies conducted in other parts of Ghana have shown similar protective effect with respect to reduced asexual parasite density in both heterozygous and homozygous α–thalassaemia carriers [20, 24]. This observation was not found in adults, as adults carrying the heterozygous α-thalassaemia were found to be more likely to harbour high asexual parasite densities, compared to the other groups. Adults have been shown to have acquired partial immunity against *P. falciparum* infections and are therefore able to clear parasites effectively due to repeated exposure [25]. We can therefore hypothesize that asymptomatic adults seem to have higher parasite threshold due to antidisease immunity, whiles children are less likely to harbour such levels of parasites without succumbing to clinical malaria. The mechanism by which the trait predisposes these adults in this study to asexual parasite carriage is not clear.

Interestingly, some genetic traits such as sickle cell traits with HbS and HbC variants that protects against severe malaria have been suggested to promote gametocyte carriage [1, 2]. However, in this study α-thalassaemia trait was not associated with increased risk of gametocyte carriage. Further studies are needed to elucidate the possible mechanisms by which these haemoglobin variants either protect against or confer susceptibility to *P. falciparum* asexual parasitaemia or gametocytaemia.

## Conclusion

The study findings showed that children with heterozygous α-thalassaemia trait had reduced risk of asexual parasite carriage under the dominant model. However, there was no association between α-thalassaemia trait and risk of gametocyte carriage, perhaps due to limited gametocyte prevalence. Further studies need to be conducted in different transmission areas to ascertain a probable association and mechanisms of α-thalassaemia and gametocyte carriage and possibly the infectiousness of these parasites in malaria transmission.

## Limitations

The very low numbers of both asexual parasites and gametocytes is an important limitation of the study and the findings described will need to be confirmed with a larger sample size.

## Abbreviations

RNA: ribonucleic acid
RT-PCR: real time reverse transcription polymerase chain reaction
